# Pickleball-related injuries treated at a tertiary academic center over five years: a cross-sectional study

**DOI:** 10.1186/s40621-026-00673-6

**Published:** 2026-04-14

**Authors:** Yue Meng, Aaron Chen, Chantal Nguyen, Matt Kaufman, Daniel Li, Nicole Pham, Raymond Chou, Eugene Roh

**Affiliations:** 1 PM&R, Department of Orthopedic Surgery, Stanford University, Redwood City, CA USA; 2 Renaissance School of Medicine at Stony Brook University, Stony Brook, NY USA; 3 Pacific Northwest University of Health Sciences College of Osteopathic Medicine, Yakima, WA USA; 4 Sports Medicine, PM&R, Stanford University, Redwood City, CA USA

**Keywords:** Pickleball, Musculoskeletal injury, Injury prevention

## Abstract

**Background:**

Pickleball has grown rapidly in popularity in recent years, accompanied by an increasing number of reported injuries among players. Our aim is to determine the epidemiology of pickleball-related injuries at a single academic center and evaluate patient-specific factors such as incidence and type of injury, mechanisms of injury, and treatment outcomes.

**Methods:**

This is a cross-sectional study at a tertiary academic outpatient orthopedic and physical medicine and rehabilitation clinic. We reviewed 164 cases of patients presenting with pickleball-related injuries from 2019 to 2023 involving the shoulder (*n* = 23), elbow (*n* = 8), wrist/hand (*n* = 30), hip/thigh (*n* = 9), knee (*n* = 52), foot/ankle (*n* = 32), and spine (*n* = 10). Independent variables included age, gender, and hand dominance. Outcome measures included injury type, laterality, treatment modality, and follow-up duration. Demographic and epidemiologic data were analyzed, and comparisons between injury characteristics were performed using appropriate statistical tests (Fisher’s exact tests and Kruskal-Wallis tests).

**Results:**

The most common pickleball-related injuries included lateral epicondylitis at the elbow (75%), rotator cuff tears at the shoulder (70%), distal radius fractures after a fall at the hand/wrist (60%), Achilles tendon tears at the foot/ankle (50%), radicular pain and spinal stenosis at the spine (50% each), medial meniscus tears at the knee (48%), and hamstring strain or rupture and iliopsoas tendinitis at the hip/thigh (33% each).Rates of injury were similar between male and female players except in the hand/wrist, which was higher among female players (77%). Non-paddle side injuries in the upper extremity occur disproportionally higher in the hand/wrist (52%) when compared to the shoulder (7%) or elbow (17%) (*p* < 0.01). There was no significant difference in laterality for lower extremity and spine injuries.

**Conclusions:**

As pickleball has become the fastest growing sport in America, the incidence of pickleball-related injuries has risen dramatically. Characterizing the wide spectrum of musculoskeletal injuries unique to pickleball may inform athletes on injury prevention considerations and allows for targeting modifiable risk factors.

## Background

Pickleball was developed by Joel Pritchard, Bill Bell, and Barney McCallums in 1965 in the United States as a summer pastime for their children [1]. The new sport used homemade table tennis equipment played approximately with tennis technique on a badminton court [2]. Players now use composite paddles akin to an oversized table tennis paddle to hit a light plastic wiffle ball back and forth across the net.

According to the Sports & Fitness Industry Association, pickleball is the fastest-growing sport in the United States since 2021. Estimated player numbers have increased from 4.8 million in 2021 to 13.6 million in 2023, approaching the popularity of table tennis at 15.4 million total players [3]. At the center of this popularity is the older demographic of Americans, with those over age 55 accounting for 40% of all players and 52% of core players, defined as those playing pickleball 8 or more times per year [4].

Part of pickleball’s broad appeal stems from its equipment and rules. Underhand serves with large paddles and a predictably bouncy ball make the game faster to learn for inexperienced or older players [5]. The “kitchen”, a non-volley zone, discourages high-velocity returns, or smashes, and serves to encourage a slower speed of play, making play easier for less athletic players [6]. As a result, pickleball is a burgeoning recreational sport suitable for players of all ages and abilities.

With this popularity, however, pickleball related injuries in the senior population have seen an 11-fold increase between 2010 and 2019 [7]. Studies have found pickleball-related injuries are increasing and that the vast majority are accounted for by Americans over 55 [8, 9]. This is thought to be due to the vulnerability of older persons to sports injuries or sudden increases in activity by those previously sedentary [5].

The currently published literature has identified important trends in pickle ball injury primarily by querying the U.S. Consumer Product Safety Commission’s National Electronic Injury Surveillance System and have queried epidemiological information obtained in the emergency department setting [7–11]. A pair of studies by Kasper et al. and Opara et al. have begun to address further characteristics of the injuries by describing upper and lower extremity pickleball injuries seen at a single center and their most common treatments [12, 13]. We hope to add to the existing literature by examining various aspects of pickleball-related musculoskeletal injuries, such as trends between player-specific factors, mechanisms of injury, and patient outcomes.

With the popularity of pickleball continuing to rise among a diverse range of players, it is vital to understand the player-specific characteristics that may affect risk of injury or the recovery course. The purpose of this study was to evaluate the epidemiology of pickleball-related upper and lower extremity injuries as well as to elucidate injury biomechanics to inform recommendations for safer play and injury prevention.

## Methods

### Subjects

This was a retrospective chart review of pickleball-associated injuries at a single academic center. Inclusion criteria were (1) patients age ≥ 18 with (2) an encounter with orthopedic subspecialties or orthopedic PM&R clinics at Stanford Healthcare outpatient center and satellite sites occurring in the 5-year period between 1/1/2019 to 12/31/2023, correlating with the sharpest recent rise in pickleball popularity (Table 1) [3]. Patients with an encounter not related to pickleball (e.g. “pickleball” was listed in the chart solely as a hobby), patients whose injury was not clearly associated to pickleball (e.g. patient did not sustain the particular injuries while playing or immediately after playing pickleball), patients with non-musculoskeletal complaints, and other unrelated cases were excluded.

**Table 1:** Search strategy employed using the STAnford Research Repository (STARR) tools

Search Strategy Items	Search Strategy Details
Used Key words	Clinical documents containing keywords “Pickleball” OR “Pickle” AND body part (e.g., shoulder, elbow, wrist, hand) in the same document
Inclusion Criteria	Age ≥ 18 Encounter with Orthopedic subspecialties or Ortho PM&R at Stanford outpatient center and satellite sites (e.g., Emeryville, Los Gatos, Pleasanton)
Exclusion Criteria	None applied during search (cases were manually excluded as described)
Time Filter	01/01/2019 to 12/31/2023

### Procedures and tools

**Fig. 1:**
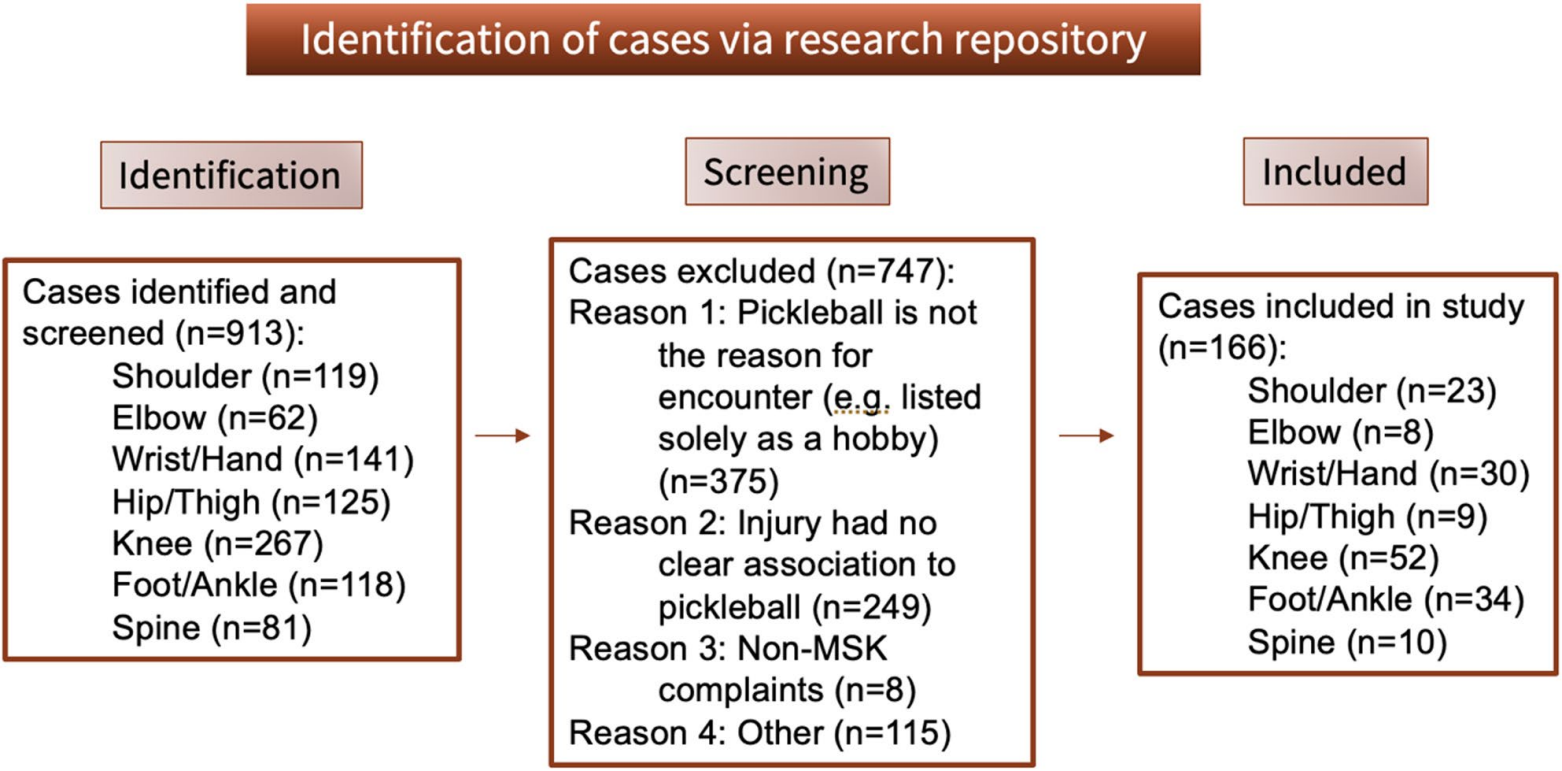
Flowchart detailing the identification of cases via the research repository. A total of 913 cases were identified and screened, with 749 of those excluded for reasons detailed in the chart. A total of 164 cases were included in the final study analysis

For each body part, cases were searched using the keywords “pickleball” or “pickle” and the body part (e.g. shoulder, elbow, wrist, hand) in the same clinical encounter documentation using STAnford Research Repository (STARR) tools (Fig. 1). Each injury was assigned with a unique and random number, with no patient identifying information such as name or MRN being collected. Approval was received from the Stanford University institutional review board prior to case identification and review.

### Outcome measures and analysis

Cases were reviewed by three resident physicians (Y.M., C.N., and M.K.) and a fourth-year medical student (A.C.) to determine that each case represents a pickleball-related injury of each body part of interest. For each case, a chart review was performed to gather specific clinical information on the injury. Demographic information collected for each case includes age of the patient at the time of injury occurrence, gender, month/year of the injury, hand dominance, and pertinent past medical history. Injury data collected for each includes laterality affected for peripheral joint injuries and spinal level for spine injuries, acuity, imaging results, length of follow up period, final diagnosis, treatment received, any history or resulting diagnosis of osteoporosis, whether the patient was able to resume playing pickleball, and any documentation of biomechanical factors resulting in the injury.

### Statistical analysis

Numerical data on patient demographics and injury epidemiology were calculated on Microsoft Excel and presented in the format of “mean (standard deviation)”, “median (minimal and maximal range)”, or “number (percentage)” depending on the data type. Fisher’s exact tests were run to analyze differences in categorical response rates among injury location groups, while Kruskal-Wallis tests were run to analyze the differences in continuous distributions among injury location groups. All analyses were run in RStudio version 2022.12.0 + 353 (Boston, MA, USA) using a two-sided level of significance of 0.05.

## Results

A total of 913 cases were identified using the STARR tools, as shown in Fig. 1. After screening and excluding cases, a total of 164 injuries were included in the final analysis.

### Overall trend

**Fig. 2:**
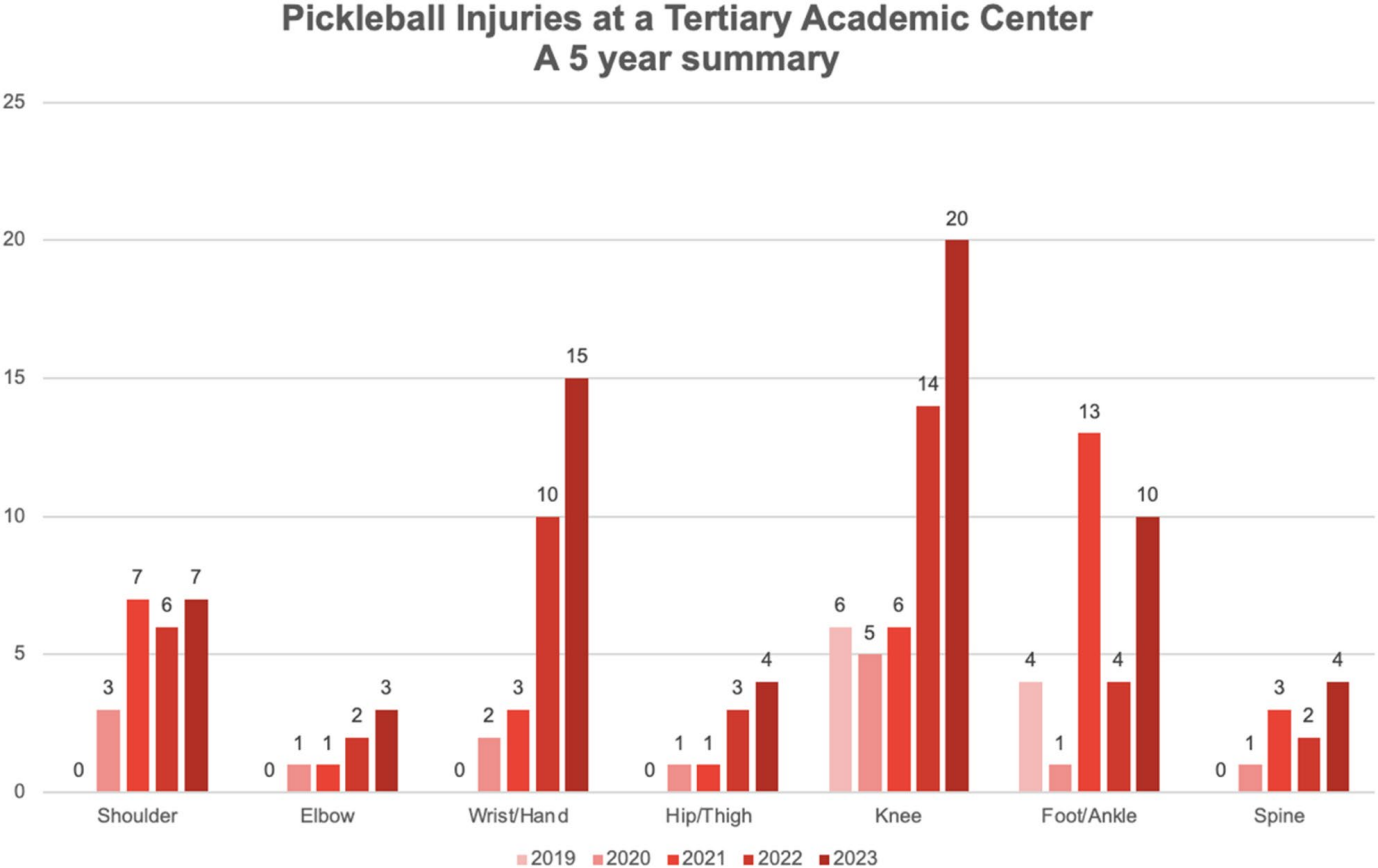
Number of injuries by year, by injured body part, from 2019 to 2023

There has been an increase in the number of pickleball injuries seen at the tertiary academic center over the five years (Fig. 2). There were 10 total injuries identified in 2019, which grew to 59 injuries in 2023. This trend is most notable for wrist/hand injuries increasing from 0 injuries in 2019 to 15 in 2023 and knee injuries increasing from 6 to 20.

### Shoulder

Twenty-three patients experienced acute shoulder injuries due to pickleball participation (Table 2). The mean age of presentation was 67, with 65% affecting male patients. 87% occurred on the paddle side of the patients. The most common pickleball-related shoulder injury was rotator cuff tear (70%), followed by rotator cuff strain (26%), shoulder impingement (26%), biceps tear (22%), superior labrum anterior-posterior (SLAP) lesion (13%), and biceps tendonitis (4%). The average follow-up duration was 12.9 months. 78% of patients received conservative management including physical therapy (PT) and injections (17%). 35% of the patients received surgery. The most mentioned injury mechanism was pain felt during hitting the ball overhead. Other mechanisms included shoulder pain resulting from a fall onto the upper extremity, as well as acute pain and deformity felt during either a forehand or a backhand shot.

**Table 2:** Demographic and injury summary of pickleball-related upper extremity injuries

Shoulder (*n* = 23)		Elbow (*n* = 8)		Hand/Wrist (*n* = 30)	
Variable	Number	Variable	Number	Variable	Number
**Gender**		**Gender**		**Gender**	
Female	8 (34.8%)	Female	6 (75%)	Female	23 (76.7%)
Male	15 (65.2%)	Male	2 (25%)	Male	7 (23.3%)
**Presentation Age**		**Presentation Age**		**Presentation Age**	
Mean (SD)	65.8 (7.92)	Mean (SD)	64.8 (7.17)	Mean (SD)	63.5 (12.4)
Median [Min, Max]	67.0 [44.0, 81.0]	Median [Min, Max]	63.0 [54.0, 80.0]	Median [Min, Max]	67.0 [24.0, 85.0]
**Side affected**		**Side affected**		**Side affected**	
Paddle side	13 (56.5%)	Paddle side	5 (62.5%)	Paddle side	14 (46.7%)
Non-paddle side	1 (4.3%)	Non-paddle side	1 (12.5%)	Non-paddle side	15 (50.0%)
Unknown	9 (39.1%)	Unknown	2 (25.0%)	Unknown	1 (3.3%)
**Follow-Up Duration (months)**		**Follow-Up Duration (months)**		**Follow-Up Duration (months)**	
Mean (SD)	12.0 (10.9)	Mean (SD)	6.50 (4.12)	Mean (SD)	3.87 (3.31)
Median [Min, Max]	10.0 [0, 36.0]	Median [Min, Max]	6.00 [3.00, 11.0]	Median [Min, Max]	3.00 [0, 12.0]
**Diagnosis**		**Diagnosis**		**Diagnosis**	
Rotator Cuff Tear	16 (69.6%)	Lateral Epicondylitis	6 (75.0%)	Distal Radius Fracture	18 (60.0%)
Impingement	6 (26.1%)	Distal Humerus Fracture	2 (25.0%)	Scaphoid Fracture	2 (6.7%)
Rotator Cuff Strain	6 (26.1%)			Metacarpal Fracture	2 (6.7%)
Biceps Tear	5 (21.7%)			TFCC Sprain	2 (6.7%)
SLAP Lesion	3 (13.0%)			Finger Dislocation	2 (6.7%)
Biceps Tendinitis	1 (4.3%)			Phalanx Fracture	1 (3.3%)
				Ulnar fracture	1 (3.3%)
				FCU tendinitis	1 (3.3%)
				Radial tunnel	1 (3.3%)
				Mallet finger	1 (3.3%)
				Contusion	1 (3.3%)
**Treatment**		**Treatment**		**Treatment**	
Conservative/PT	18 (78.3%)	Conservative/PT	6 (75.0%)	Conservative/PT	23 (76.7%)
Injection	4 (17.4%)	Injection	2 (25.0%)	Injection	0 (0%)
Surgery	8 (34.8%)	Surgery	3 (37.5%)	Surgery	9 (30%)

### Elbow

Eight cases of elbow injuries were identified, with 75% of which diagnosed as lateral epicondylitis, and 25% distal humerus fracture resulting from a fall. 75% were female and 25% were male. The mean age of presentation was 65. Out of the cases with known laterality, 83% occurred on the paddle side of the patients. The mean follow-up duration was 6.5 months. 75% of the elbow patients were treated with conservative management including PT, and 38% patients ultimately received surgery (e.g., open reduction and internal fixation [ORIF] for elbow fracture).

### Hand/wrist

A total of 30 cases of acute, pickleball-related wrist and/or hand injuries were included in our analysis (Table 2). The mean age was 65 years, with 77% female and 23% male. The most prevalent injury was distal radius fracture affecting 60% of patients, followed by finger dislocation, triangular fibrocartilage complex (TFCC) sprain, scaphoid fracture, and metacarpophalangeal (MCP) fracture, each affecting 6% of patients. Most of the injuries resulted from fall while running to reach the ball.

Paddle-sided injuries (48%) occurred at about the same rate as non-paddle-sided injuries (53%). 70% of patients received conservative treatment including immobilization with cast or buddy tape, or occupational/hand therapy (OT). Surgical treatment with ORIF was performed on 30% of the cases.

**Table 3:** Demographic and injury summary of pickleball-related lower extremity and spinal injuries

	Hip/Thigh		Knee		Foot/Ankle		Spine
	(*N* = 9)		(*N* = 52)		(*N* = 34)		(*N* = 10)
**Gender**		**Gender**		**Gender**		**Gender**	
Female	5 (56%)	Female	24 (46%)	Female	18 (53%)	Female	5 (50%)
Male	4 (44%)	Male	28 (54%)	Male	16 (47%)	Male	5 (50%)
**Presentation Age**		**Presentation Age**		**Presentation Age**		**Presentation Age**	
Mean (SD)	66 (17)	Mean (SD)	54 (14)	Mean (SD)	53 (15)	Mean (SD)	58 (15)
Median [Min, Max]	71 [27, 83]	Median [Min, Max]	55 [21, 77]	Median [Min, Max]	56 [27, 85]	Median [Min, Max]	63 [25, 73]
**Side affected**		**Side affected**		**Side affected**		**Side affected**	
Bilateral	0 (0%)	Bilateral	1 (2%)	Unknown	1 (3%)	Bilateral	1 (10.0%)
Right	4 (44%)	Right	27 (52%)	Right	20 (59%)	Right	2 (20.0%)
Left	5 (56%)	Left	24 (46%)	Left	13 (38%)	Left	2 (20.0%)
**Follow-Up Duration (months)**		**Follow-Up Duration (months)**		**Follow-Up Duration (months)**		**Follow-Up Duration (months)**	
Mean (SD)	9.1 (11.3)	Mean (SD)	8.5 (11.4)	Mean (SD)	9.90 (13.1)	Mean (SD)	3.9 (5.3)
Median [Min, Max]	4.0 [1.0, 32.0]	Median [Min, Max]	4.0 [0, 56.0]	Median [Min, Max]	6.50 [0, 60.0]	Median [Min, Max]	2.0 [0, 17.0]
**Diagnosis**		**Diagnosis**		**Diagnosis**		**Diagnosis**	
Hamstring Strain/Rupture	3 (33%)	Medial Meniscal Tear	25 (48%)	Achilles Tear	16 (47%)	Radiculitis/radiculopathy/radicular pain	5 (50.0%)
Iliopsoas Tendinitis	3 (33%)	Lateral Meniscal Tear	10 (19%)	Achilles Tendinitis	4 (12%)	Stenosis	5 (50.0%)
Labral Tear	2 (22%)	Knee Osteoarthritis	8 (15%)	Ankle Sprain	4 (12%)	Discogenic pain	2 (20.0%)
Femur Fracture	2 (22%)	ACL sprain	4 (8%)	Calf Strain	2 (6%)	Fracture of cervical vertebrae	1 (10.0%)
IT Band Syndrome	1 (11%)	ACL tear	4 (8%)	Bone Reaction, Stress Fx	2 (6%)	Lumbar strain	1 (10.0%)
GTPS	1 (11%)	Patellar Chondromalacia/Tendinitis	4 (8%)	Arthritis	1 (3%)	Other	1 (10.0%)
		Patellofemoral Arthritis	4 (8%)	Peroneal Tendinopathy	1 (3%)	Facetogenic pain	1 (10.0%)
		Posttraumatic Arthritis	3 (6%)	Lisfranc Tear	1 (3%)		
		Complication of prior surgery	3 (6%)	Fracture	1 (3%)		
		Patellofemoral Syndrome	2 (4%)	Plantar Fasciitis	1 (3%)		
		Tibial Plateau Fracture	2 (4%)	Plantar Fascia Tear and Tibial Tunnel Syndrome	1 (3%)		
		Quadriceps Tendon Rupture	1 (2%)				
		Patellar Fracture	1 (2%)				
		Patellar Subluxation	1 (2%)				
		Saphenous Nerve Compression	1 (2%)				
**Treatment**		**Treatment**		**Treatment**		**Treatment**	
Conservative/PT	7 (78%)	Conservative/PT	37 (71%)	Conservative/PT	19 (56%)	Conservative/PT	9 (90%)
Injection	2 (22%)	Injection	13 (25%)	Injection	2 (6%)	Injection	4 (40%)
Surgery	2 (22%)	Surgery	23 (44%)	Surgery	15 (44%)	Surgery	1 (10%)

### Hip/thigh

We identified 9 cases of acute onset hip/thigh injuries specifically related to participation in sport (Table 3). The mean age was 66 years, with 56% female and 44% male. The most prevalent diagnosis was tendinopathy, affecting 67% patients with a specific predilection for proximal hamstring (22%), iliopsoas (22%), gluteus medius (11%), and iliotibial band (11%). Some patients with these tendinopathies had concomitant hip labral tears (22%) upon further MRI imaging. A smaller subset of patients, typically those who were older and female, had femoral neck fractures from falls while playing pickleball (22%), though these patients had no known history of osteoporosis or osteopenia.

Management options for thigh/hip injuries from acute pickleball-induced injuries included 78% conservative management, out of which 2 patients with iliopsoas tendinopathy/bursitis pursued injections. Surgical management was pursued for those with acute fractures (22%).

### Knee

Fifty-two patients had acute knee injuries while playing pickleball. The mean age was 54 years, with 54% male and 46% female. 52% of injuries occurred to players’ right knee (52%), 46% to the left knee, and 2% affecting both knees. The mean follow-up duration was 8.5 months. The most prevalent pickleball-induced knee injuries were meniscal tears, accounting for 67% of observed knee injuries. More injuries affected the medial meniscus (48% of injuries) than the lateral meniscus (19% of injuries). The next most prevalent diagnoses were knee osteoarthritis (15% of injuries) and patellofemoral arthritis (8%), patellar chondromalacia/tendinitis (8%), anterior cruciate ligament (ACL) tear (8%), and ACL sprain (8%). 71% of these injuries were at least initially managed conservatively with relative rest, physical therapy, over-the-counter medications, and/or injections. 44% of the patients eventually underwent surgical intervention.

### Ankle/foot

Thirty-four patients were identified as having an acute foot and/or ankle injury related to playing pickleball. Mean age was 53 years, with 53% female and 47% male. Twenty injuries occurred on the right side (59%) as compared to 13 on the left side (38%). The mean length of follow up was 9.9 months. The most prevalent injury within the group was a complete Achilles tear (50%). The next most common injuries were Achilles tendinopathy or an ankle sprain which accounted for 12% each. Other diagnoses include tendinopathies, stress fractures, arthritis, calf strains, and plantar fascia injuries. The treatment method was dependent on the pathology, with all 16 patients with Achilles tears opting for surgery (one was cancelled due to pre-operative deep venous thrombosis). The remaining patients were mostly managed conservatively, including PT, durable medical equipment (most often a heel lift or controlled ankle motion [CAM] boot), injections (2 patients) and shockwave therapy (1 patient).

The Achilles tear group was younger with a mean age at 49, and mostly male (81%). The most commonly reported mechanism of injury was an explosive maneuver such as running after a ball, lunging, or pushing off. Of the 12 for which returntosport data was collected, 10 were cleared to return to activity without modifications.

### Spine

Ten patients presented with acute complaints of back or neck pain. The mean age was 58 years, with equal numbers of males (50%) and females (50%) in this population. Most injuries occurred at the lumbar spine (80%), followed by the cervical spine (10%). The mean follow-up duration was 3.9 months. The most prevalent pickleball-related spine injury diagnoses were radicular pain (50% of injuries), spinal stenosis (50%), discogenic pain (20%), followed by cervical spine fracture, lumbar strain, facetogenic pain, degenerative disc disease and degenerative scoliosis (each 10%). 90% were managed conservatively with rest/PT and oral medications. Four patients pursued an injection (3 transforaminal epidural injections and 1 facet joint injection), and the one acute fracture was managed with surgery. Although there was limited information on injury mechanisms, two separate patient encounters noted pain with repeated twisting motions during pickleball.

### Comparison between injury groups

For the upper extremity, elbow and hand/wrist injuries have significantly higher percentage of female players than patients with shoulder injuries (*p* < 0.01) (Table 4).

**Table 4:** Differences in demographic results, injury types, and outcome among upper extremity injury location groups with p-values indicated

Shoulder (*n* = 23)		Elbow (*n* = 8)		Hand/Wrist (*n* = 30)		*p*-value
**Gender**		**Gender**		**Gender**		
Female	8 (34.8%)	Female	6 (75%)	Female	23 (76.7%)	0.006**
Male	15 (65.2%)	Male	2 (25%)	Male	7 (23.3%)	
**Side affected**		**Side affected**		**Side affected**		
Paddle side	13 (56.5%)	Paddle side	5 (62.5%)	Paddle side	14 (46.7%)	
Non-paddle side	1 (4.3%)	Non-paddle side	1 (12.5%)	Non-paddle side	15 (50.0%)	0.006**
Unknown	9 (39.1%)	Unknown	2 (25.0%)	Unknown	1 (3.3%)	
**Follow-Up Duration (months)**		**Follow-Up Duration (months)**		**Follow-Up Duration (months)**		
Mean (SD)	12.0 (10.9)	Mean (SD)	6.50 (4.12)	Mean (SD)	3.87 (3.31)	0.014*
Median [Min, Max]	10.0 [0, 36.0]	Median [Min, Max]	6.00 [3.00, 11.0]	Median [Min, Max]	3.00 [0, 12.0]	

* indicates p-value of < 0.05, ** indicates p-value of < 0.01

Further, there are significantly more non-paddle-sided injuries in the wrist than in the elbow or shoulder (*p* < 0.01), and wrist fractures were significantly more common on the non-paddle side (*p* < 0.01). Lastly, average follow-up duration was significantly longer for shoulder injuries than other upper extremity injuries (*p* < 0.05).

**Table 5:** Differences in demographic results and treatment types among lower extremity and spinal injuries with p-values indicated

	Foot/Ankle		Knee		Spine		Hip/Thigh	*p*-value
	(*N* = 34)		(*N* = 52)		(*N* = 10)		(*N* = 9)	
**Presentation Age**		**Presentation Age**		**Presentation Age**		**Presentation Age**		
Mean (SD)	53.3 (14.7)	Mean (SD)	53.8 (13.5)	Mean (SD)	58.1 (14.9)	Mean (SD)	65.9 (17.2)	0.038*
Median [Min, Max]	55.5 [27.0, 85.0]	Median [Min, Max]	55.0 [21.0, 77.0]	Median [Min, Max]	62.5 [25.0, 73.0]	Median [Min, Max]	71.0 [27.0, 83.0]	
**Treatment**		**Treatment**		**Treatment**		**Treatment**		
Conservative/PT	17 (50.0%)	Conservative/PT	37 (71.2%)	Conservative/PT	9 (90%)	Conservative/PT	5 (55.6%)	0.065
Injection	2 (5.9%)	Injection	13 (25.0%)	Injection	4 (40%)	Injection	2 (22.2%)	0.030*
Surgery	15 (44.1%)	Surgery	23 (44.2%)	Surgery	1 (10%)	Surgery	2 (22.2%)	0.135

* indicates p-value of < 0.05, ** indicates p-value of < 0.01

For the lower extremity and spine, patients with hip/thigh injuries were older at presentation compared to patients of other injury locations (*p* < 0.05) (Table 5). There was a higher percentage of patients with spine injuries receiving injections as part of the treatment, compared to patients with other injured locations (*p* < 0.05). There were no statistically significant differences in the gender, laterality, or follow-up duration found amongst lower extremity and spine injury cohorts.

## Discussion

This 5-year retrospective analysis of all pickleball-related injuries treated at a single tertiary academic sports center demonstrated increasing rates of pickleball-related injuries requiring medical or surgical treatment.

In the upper extremity, the arm using the paddle experienced more overuse injuries, whereas the contralateral arm had a higher rate of injury from falls. Overuse injuries in the shoulder may occur due to overhead shots, and in the elbow with repetitive swinging motion. In our cohort, the high rate of wrist fractures suffered on the non-paddle side during falls may eflect a tendency for players to brace themselves with the hand not holding the paddle.

Injuries of the lower extremity and spine primarily were related to overuse and did not show a significant difference in laterality, age, or gender. Tendinopathies in the thigh and hip may occur with sudden motion such as a lunge to reach the ball or run to the kitchen line. Knee meniscal injuries can occur from twisting or pivoting motions while attempting to reach the ball. Foot and ankle injuries were most commonly reported when players pushed off one foot to reach the ball or ran toward the kitchen line. Ankle sprains were frequently associated with a mechanism of foot inversion. Low back pain may occur with repeated twisting motion of the spine.

In Table 6, we propose possible injury prevention strategies for each injury based on the mechanisms of injury in our patient. For example, as rotator cuff tears occurred most frequently secondary to overhead shots, we recommend targeted muscle strengthening exercises and play modification limiting overhead motions until symptoms improve. With many injuries occurring as a result of fall during pickleball, proper footwear as well as off-the-court training in proprioception, balance, and coordination are also crucial in fall prevention and play optimization.

**Table 6:** Proposed injury mechanism and preventive strategies by anatomic location

Anatomic region and injury	Possible Mechanism	Preventive strategies
Shoulder		
Rotator cuff tears	Overhead shots, falls	Strengthen rotator cuff, avoid overheads, fall prevention, scapular stabilization [14]
Impingement	Repetitive overhead motions	Strengthening rotator cuff muscles, biceps, and scapulothoracic muscles, avoid overheads [14]
Biceps tear	Rotational swinging motion	Warm up, avoid sudden increase in intensity
Elbow		
Lateral epicondylitis	Backhand shots/tight grip	Learn proper technique, appropriate grip size, use two hands, strengthening forearm muscles
Wrist		
Wrist fractures	FOOSH	Fall prevention
TFCC sprain	Shots with ulnar deviation (e.g. roll volleys)	Learn proper technique, use two hands
Hip/Thigh		
Iliopsoas tendinopathy/bursitis	Repetitive hip flexion	Stretching and strengthening of hip adductors, abductors, extensors, and flexors starting with eccentric and isometric exercises; avoid sudden increases in intensity [15]
Gluteus medius/ proximal hamstring tendinopathy	Sudden hip abduction/knee flexion when lunging	
IT band tendinopathy	Running, worn out shoes, quick change of direction	
Knee		
Meniscal tears	Twisting or pivoting while planting foot	Correct any muscle strength imbalances, i.e. quadriceps vs. hamstrings [16]; avoid twisting knee motion on a planted foot; incorporate multicomponent training programs (e.g. agility, balance, and flexibility) [17]
ACL injuries	Lunging, jumping with twisting motion, awkward landing	
OA, patellofemoral arthritis	Overuse, low grade trauma	Quadriceps/vastus medialis oblique strengthening, McConnell taping
Foot/Ankle		
Achilles tear	Pushing off, rapid lunge	Intrinsic foot/ankle muscle strengthening, stretching, wear footwear with good heel support, minimize rapid increases in training, avoid playing when overly fatigued
Ankle sprain	Twisted ankle/inversion	
Spine		
Radicular pain, discogenic, stenosis	Pre-existing spinal conditions, twisting motion	Core strengthening

This study has several limitations to consider when interpreting the results. Though five years of cases were included, the total number of identified cases resulted in a small sample size that limited further analyses beyond descriptive statistics. Future multicenter investigations or studies with larger, population-based cohorts are needed to provide a more comprehensive understanding of the full injury spectrum. Further, since the research repository only includes patients who sought care at a tertiary medical center outpatient clinics, our findings likely do not capture the full spectrum of pickleball-related injuries, particularly those that are either more minor and may not prompt evaluation by a specialist or more severe injuries that may present directly to an emergency department or acute care hospital. In addition, we recognize that certain injuries, such as distal radius fractures in older adults, may serve as indicators of underlying osteoporosis; however, given the limited bone health data available in our cohort, further studies are needed to better evaluate this relationship and its potential implications for screening. Lastly, as a retrospective chart review, collected data was limited to what was documented in the clinical encounter, which often lacked players’ skill levels or pickleball experience. Despite these limitations, however, the study provides insight into common pickleball-related injuries and offers direction for future studies about pickleball-related injuries, mechanisms, and preventative measures.

## Conclusions

As pickleball has become the fastest growing sport in America, the incidence of pickleball-related injuries has risen dramatically. This study provides valuable single-center epidemiologic data on pickleball-related injuries and evaluates the influence of patient-specific factors on injury incidence and type. A combination of fall prevention techniques, off-the-court training in proprioception, balance, coordination, muscle strengthening exercises, and proper form targeted to certain shots in pickleball can likely help players mitigate injury risks in playing pickleball.

## Data Availability

The datasets generated and/or analyzed during the current study are not publicly available due to institutional and privacy restrictions but are available from the corresponding author on reasonable request.

## Abbreviations

ACL: Anterior cruciate ligament
CAM: Controlled ankle motion
FOOSH: Fall onto outstretched hand
MCP: Metacarpophalangeal
ORIF: Open reduction and internal fixation
PT: Physical therapy
OT: Occupational therapy
SLAP: Superior labrum anterior-posterior
STARR: STAnford Research Repository
TFCC: Triangular fibrocartilage complex
